# Bis{2-[(phenyl­imino)­meth­yl]-1*H*-pyrrol-1-ido}palladium(II)

**DOI:** 10.1107/S1600536812048143

**Published:** 2012-11-30

**Authors:** Wolfgang Imhof

**Affiliations:** a University Koblenz-Landau, Institute for Integrated Natural Sciences, Universitätsstrasse 1, 56070 Koblenz, Germany

## Abstract

In the title complex, [Pd(C_11_H_9_N_2_)_2_], the Pd^II^ atom is located on an inversion centre and has a square-planar coordination geometry. The phenyl substituents at the imine N atoms make a dihedral angle of 75.0 (6)° with respect to the PdN_4_ plane.

## Related literature

For structure analyses of the free ligand *N*-[(1*H*-pyrrol-2-yl)methyl­ene]aniline, see: Gomes *et al.* (2010 ▶); Crestani *et al.* (2011 ▶). For the structure of a related nickel complex of the same imine ligand and an additional bipyridine ligand, see: Castro *et al.* (1992 ▶). For the structure of a related palladium complex with a different aromatic substituent, see: Liang *et al.* (2004 ▶).
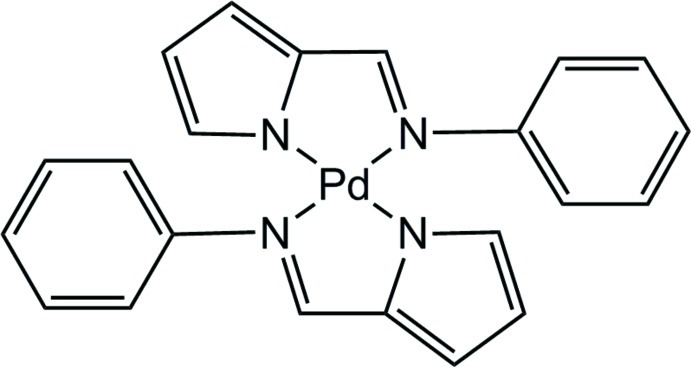


## Experimental

### 

#### Crystal data

[Pd(C_11_H_9_N_2_)_2_]
*M* *_r_* = 444.80
Monoclinic, 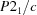

*a* = 10.5634 (4) Å
*b* = 10.6480 (6) Å
*c* = 8.0560 (7) Å
β = 93.044 (2)°
*V* = 904.85 (10) Å^3^
*Z* = 2
Mo *K*α radiation
μ = 1.04 mm^−1^
*T* = 183 K
0.60 × 0.10 × 0.02 mm

#### Data collection

Nonius KappaCCD diffractometer
3903 measured reflections
2018 independent reflections
1464 reflections with *I* > 2σ(*I*)
*R*_int_ = 0.035

#### Refinement

*R*[*F* ^2^ > 2σ(*F* ^2^)] = 0.032
*wR*(*F* ^2^) = 0.078
*S* = 1.00
2018 reflections
124 parameters
H-atom parameters constrained
Δρ_max_ = 0.31 e Å^−3^
Δρ_min_ = −0.75 e Å^−3^

### 

Data collection: *COLLECT* (Nonius, 1998 ▶); cell refinement: *DENZO* (Otwinowski & Minor, 1997 ▶); data reduction: *DENZO*; program(s) used to solve structure: *SHELXS97* (Sheldrick, 2008 ▶); program(s) used to refine structure: *SHELXL97* (Sheldrick, 2008 ▶); molecular graphics: *ORTEP-3* (Farrugia, 2012 ▶); software used to prepare material for publication: *publCIF* (Westrip, 2010 ▶).

## Supplementary Material

Crystal structure: contains datablock(s) I, global. DOI: 10.1107/S1600536812048143/su2533sup1.cif

Structure factors: contains datablock(s) I. DOI: 10.1107/S1600536812048143/su2533Isup2.hkl

Additional supplementary materials:  crystallographic information; 3D view; checkCIF report

## Figures and Tables

**Fig. 1.**
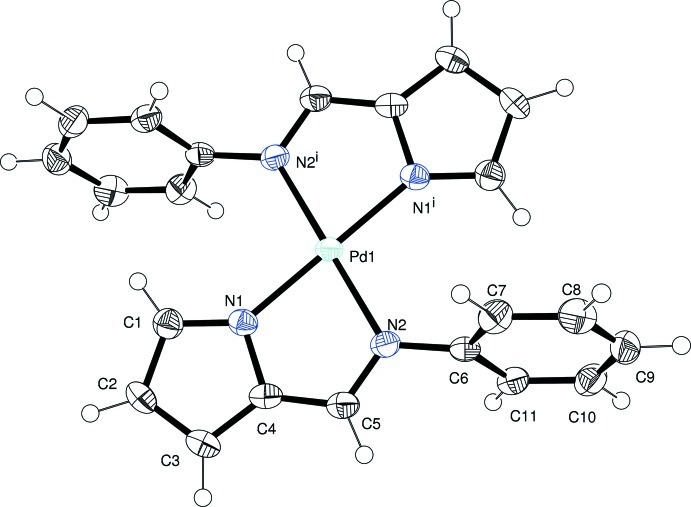
Molecular structure of the title compound with the atom numbering. The displacement ellipsoids are drawn at the 50% probability level (symmetry code: (i) = -x+1, -y+1, -z).

## supplementary crystallographic information

### Comment

In the course of a project related to the supramolecular structures of square planar nickel and palladium complexes of pyrrole-2-carbaldehyde based Schiff base ligands in comparison with the structures of the free ligands the molecular structure of the title compound was determined. The free ligands form inversion dimers *via* N—H···N hydrogen bonds between the pyrrole NH function and the imine nitrogen atom of a neighbouring molecule (Crestani *et al.*, 2011; Gomes *et al.* 2010).

The molecular structure of the title compound is presented in Fig. 1. The central palladium atom is located on a crystallographic inversion center. The phenyl substituents at the imine nitrogen atoms show a dihedral angle of 75.0 (6)° with respect to the PdN_4_ plane. As is expected the bond lengths in the NCCN backbone of the ligand change upon coordination to palladium. The C4–N1 bond in the pyrrole subunit is slightly elongated to 1.389 (3) Å. In addition, C4–C5 bond is shortened to 1.400 (4) Å whereas the imine double bond C5–N2 is elongated to 1.310 (4) Å.

### Experimental

*N*-((1*H*-Pyrrol-2-yl)methylene)aniline (170 mg, 1 mmol) and [Pd(PPh_3_)_4_] (580 mg, 0.5 mmol) were dissolved in 20 ml anhydrous toluene under an argon atmosphere. After the solution is stirred at room temperature for 2 h it was filtered through a short bed of celite. Afterwards the solution was concentrated to *ca.* 10 ml *in vacuo*. Yellow plate-like crystals of the title compound were obtained from this solution after 1 week at 253 K (Yield: 169 mg, 76%).

### Refinement

Hydrogen atoms were included into calculated positions and treated as riding: C-H = 0.95 Å with *U*_iso_(H) = 1.2*U*_eq_(C).

### Crystal data

**Table:**

[Pd(C_11_H_9_N_2_)_2_]	*F*(000) = 448
*M**_r_* = 444.80	*D*_x_ = 1.633 Mg m^−^^3^
Monoclinic, *P*2_1_/*c*	Mo *K*α radiation, λ = 0.71073 Å
Hall symbol: -P 2ybc	Cell parameters from 3903 reflections
*a* = 10.5634 (4) Å	θ = 2.7–27.5°
*b* = 10.6480 (6) Å	µ = 1.04 mm^−^^1^
*c* = 8.0560 (7) Å	*T* = 183 K
β = 93.044 (2)°	Plate, yellow
*V* = 904.85 (10) Å^3^	0.6 × 0.1 × 0.02 mm
*Z* = 2	

### Data collection

**Table:**

Nonius KappaCCD diffractometer	1464 reflections with *I* > 2σ(*I*)
Radiation source: fine-focus sealed tube	*R*_int_ = 0.035
Graphite monochromator	θ_max_ = 27.5°, θ_min_ = 2.7°
phi–scan, ω–scan	*h* = −13→13
3903 measured reflections	*k* = −13→13
2018 independent reflections	*l* = 0→10

### Refinement

**Table:**

Refinement on *F*^2^	Primary atom site location: structure-invariant direct methods
Least-squares matrix: full	Secondary atom site location: difference Fourier map
*R*[*F*^2^ > 2σ(*F*^2^)] = 0.032	Hydrogen site location: inferred from neighbouring sites
*wR*(*F*^2^) = 0.078	H-atom parameters constrained
*S* = 1.00	*w* = 1/[σ^2^(*F*_o_^2^) + (0.0376*P*)^2^ + ] where *P* = (*F*_o_^2^ + 2*F*_c_^2^)/3
2018 reflections	(Δ/σ)_max_ < 0.001
124 parameters	Δρ_max_ = 0.31 e Å^−^^3^
0 restraints	Δρ_min_ = −0.75 e Å^−^^3^

### Special details

**Table:**

Geometry. All e.s.d.'s (except the e.s.d. in the dihedral angle between two l.s. planes) are estimated using the full covariance matrix. The cell e.s.d.'s are taken into account individually in the estimation of e.s.d.'s in distances, angles and torsion angles; correlations between e.s.d.'s in cell parameters are only used when they are defined by crystal symmetry. An approximate (isotropic) treatment of cell e.s.d.'s is used for estimating e.s.d.'s involving l.s. planes.
Refinement. Refinement of *F*^2^ against ALL reflections. The weighted *R*-factor *wR* and goodness of fit *S* are based on *F*^2^, conventional *R*-factors *R* are based on *F*, with *F* set to zero for negative *F*^2^. The threshold expression of *F*^2^ > σ(*F*^2^) is used only for calculating *R*-factors(gt) *etc*. and is not relevant to the choice of reflections for refinement. *R*-factors based on *F*^2^ are statistically about twice as large as those based on *F*, and *R*- factors based on ALL data will be even larger.

### Fractional atomic coordinates and isotropic or equivalent isotropic displacement parameters (Å^2^)

**Table:**

	*x*	*y*	*z*	*U*_iso_*/*U*_eq_	
Pd1	0.5000	0.5000	0.0000	0.02321 (12)	
N1	0.6789 (2)	0.5646 (2)	0.0414 (3)	0.0262 (6)	
C1	0.7967 (3)	0.5447 (3)	−0.0074 (4)	0.0312 (7)	
H1	0.8188	0.4863	−0.0902	0.037*	
C2	0.8824 (3)	0.6229 (3)	0.0821 (4)	0.0315 (7)	
H2	0.9714	0.6268	0.0707	0.038*	
C3	0.8136 (3)	0.6933 (3)	0.1901 (4)	0.0321 (7)	
H3	0.8457	0.7552	0.2662	0.038*	
C4	0.6880 (3)	0.6555 (3)	0.1652 (3)	0.0285 (7)	
C5	0.5768 (3)	0.6762 (3)	0.2483 (3)	0.0299 (7)	
H5	0.5744	0.7365	0.3349	0.036*	
N2	0.4762 (2)	0.6101 (2)	0.2034 (3)	0.0270 (6)	
C6	0.3624 (3)	0.6222 (3)	0.2888 (3)	0.0280 (7)	
C7	0.3223 (3)	0.5215 (3)	0.3841 (4)	0.0339 (8)	
H7	0.3735	0.4486	0.3975	0.041*	
C8	0.2079 (4)	0.5286 (3)	0.4586 (4)	0.0385 (8)	
H8	0.1811	0.4606	0.5245	0.046*	
C9	0.1314 (3)	0.6348 (3)	0.4379 (4)	0.0395 (8)	
H9	0.0511	0.6382	0.4858	0.047*	
C10	0.1739 (3)	0.7353 (3)	0.3466 (4)	0.0363 (8)	
H10	0.1232	0.8088	0.3350	0.044*	
C11	0.2885 (3)	0.7303 (3)	0.2724 (3)	0.0300 (7)	
H11	0.3168	0.8000	0.2107	0.036*	

### Atomic displacement parameters (Å^2^)

**Table:**

	*U*^11^	*U*^22^	*U*^33^	*U*^12^	*U*^13^	*U*^23^
Pd1	0.02423 (19)	0.02118 (18)	0.02387 (18)	−0.00028 (14)	−0.00192 (12)	−0.00276 (13)
N1	0.0275 (14)	0.0234 (14)	0.0274 (12)	−0.0012 (11)	−0.0018 (11)	0.0008 (10)
C1	0.0302 (18)	0.0304 (15)	0.0328 (16)	0.0010 (14)	−0.0002 (14)	0.0008 (13)
C2	0.0240 (16)	0.0340 (17)	0.0361 (16)	−0.0078 (14)	−0.0021 (14)	0.0057 (13)
C3	0.0318 (18)	0.0316 (18)	0.0319 (16)	−0.0076 (14)	−0.0064 (14)	−0.0004 (13)
C4	0.0356 (18)	0.0226 (15)	0.0268 (14)	−0.0029 (13)	−0.0027 (14)	−0.0022 (12)
C5	0.0338 (18)	0.0272 (16)	0.0283 (15)	−0.0008 (13)	−0.0029 (14)	−0.0042 (12)
N2	0.0290 (14)	0.0246 (13)	0.0271 (13)	0.0011 (11)	−0.0009 (11)	−0.0037 (10)
C6	0.0286 (16)	0.0311 (17)	0.0238 (14)	−0.0030 (13)	−0.0024 (13)	−0.0076 (12)
C7	0.0372 (19)	0.035 (2)	0.0297 (16)	0.0033 (14)	0.0008 (14)	0.0003 (12)
C8	0.045 (2)	0.039 (2)	0.0308 (17)	−0.0062 (15)	0.0028 (16)	0.0030 (13)
C9	0.0298 (18)	0.055 (2)	0.0340 (16)	−0.0047 (16)	0.0056 (14)	−0.0123 (15)
C10	0.0348 (19)	0.039 (2)	0.0351 (17)	0.0095 (15)	−0.0014 (15)	−0.0080 (14)
C11	0.0339 (18)	0.0276 (17)	0.0281 (15)	0.0026 (14)	−0.0020 (14)	−0.0035 (12)

### Geometric parameters (Å, º)

**Table:**

Pd1—N1	2.022 (2)	C5—H5	0.9500
Pd1—N1^i^	2.022 (2)	N2—C6	1.422 (4)
Pd1—N2	2.041 (2)	C6—C11	1.393 (4)
Pd1—N2^i^	2.041 (2)	C6—C7	1.398 (4)
N1—C1	1.342 (4)	C7—C8	1.379 (5)
N1—C4	1.389 (3)	C7—H7	0.9500
C1—C2	1.401 (4)	C8—C9	1.395 (5)
C1—H1	0.9500	C8—H8	0.9500
C2—C3	1.384 (4)	C9—C10	1.387 (5)
C2—H2	0.9500	C9—H9	0.9500
C3—C4	1.391 (4)	C10—C11	1.379 (4)
C3—H3	0.9500	C10—H10	0.9500
C4—C5	1.400 (4)	C11—H11	0.9500
C5—N2	1.310 (4)		
			
N1—Pd1—N1^i^	180.0	C4—C5—H5	120.9
N1—Pd1—N2	80.00 (9)	C5—N2—C6	120.8 (2)
N1^i^—Pd1—N2	100.00 (9)	C5—N2—Pd1	113.4 (2)
N1—Pd1—N2^i^	100.00 (9)	C6—N2—Pd1	125.75 (18)
N1^i^—Pd1—N2^i^	80.00 (9)	C11—C6—C7	120.1 (3)
N2—Pd1—N2^i^	180.0	C11—C6—N2	120.9 (3)
C1—N1—C4	106.9 (3)	C7—C6—N2	119.0 (3)
C1—N1—Pd1	140.5 (2)	C8—C7—C6	119.7 (3)
C4—N1—Pd1	112.6 (2)	C8—C7—H7	120.1
N1—C1—C2	109.8 (3)	C6—C7—H7	120.1
N1—C1—H1	125.1	C9—C8—C7	120.5 (3)
C2—C1—H1	125.1	C9—C8—H8	119.7
C3—C2—C1	107.4 (3)	C7—C8—H8	119.7
C3—C2—H2	126.3	C10—C9—C8	119.1 (3)
C1—C2—H2	126.3	C10—C9—H9	120.4
C2—C3—C4	106.3 (3)	C8—C9—H9	120.4
C2—C3—H3	126.8	C9—C10—C11	121.1 (3)
C4—C3—H3	126.8	C9—C10—H10	119.4
N1—C4—C3	109.5 (3)	C11—C10—H10	119.4
N1—C4—C5	115.2 (3)	C6—C11—C10	119.4 (3)
C3—C4—C5	134.7 (3)	C6—C11—H11	120.3
N2—C5—C4	118.2 (3)	C10—C11—H11	120.3
N2—C5—H5	120.9		

Symmetry code: (i) −*x*+1, −*y*+1, −*z*.
